# Genetic Analysis of the Role of Protein Kinase Cθ in Platelet Function and Thrombus Formation

**DOI:** 10.1371/journal.pone.0003277

**Published:** 2008-09-25

**Authors:** Kellie J. Hall, Matthew T. Harper, Karen Gilio, Judith M. Cosemans, Johan W. M. Heemskerk, Alastair W. Poole

**Affiliations:** 1 Department of Physiology and Pharmacology, University of Bristol, Bristol, United Kingdom; 2 Department of Biochemistry, University of Maastricht, Maastricht, The Netherlands; University of Oldenburg, Germany

## Abstract

**Background:**

PKCθ is a novel protein kinase C isozyme, predominately expressed in T cells and platelets. PKCθ^−/−^ T cells exhibit reduced activation and PKCθ^−/−^ mice are resistant to autoimmune disease, making PKCθ an attractive therapeutic target for immune modulation. Collagen is a major agonist for platelets, operating through an immunoreceptor-like signalling pathway from its receptor GPVI. Although it has recently been shown that PKCθ positively regulates outside-in signalling through integrin α_IIb_β_3_ in platelets, the role of PKCθ in GPVI-dependent signalling and functional activation of platelets has not been assessed.

**Methodology/Principal Findings:**

In the present study we assessed static adhesion, cell spreading, granule secretion, integrin α_IIb_β_3_ activation and platelet aggregation in washed mouse platelets lacking PKCθ. Thrombus formation on a collagen-coated surface was assessed in vitro under flow. PKCθ^−/−^ platelets exhibited reduced static adhesion and filopodia generation on fibrinogen, suggesting that PKCθ positively regulates outside-in signalling, in agreement with a previous report. In contrast, PKCθ^−/−^ platelets also exhibited markedly enhanced GPVI-dependent α-granule secretion, although dense granule secretion was unaffected, suggesting that PKCθ differentially regulates these two granules. Inside-out regulation of α_IIb_β_3_ activation was also enhanced downstream of GPVI stimulation. Although this did not result in increased aggregation, importantly thrombus formation on collagen under high shear (1000 s^−1^) was enhanced.

**Conclusions/Significance:**

These data suggest that PKCθ is an important negative regulator of thrombus formation on collagen, potentially mediated by α-granule secretion and α_IIb_β_3_ activation. PKCθ therefore may act to restrict thrombus growth, a finding that has important implications for the development and safe clinical use of PKCθ inhibitors.

## Introduction

The protein kinase C (PKC) family critically regulates platelet activation. Many platelet functional responses, including secretion and aggregation are reduced or abolished by broad-spectrum PKC inhibitors and enhanced by PKC activators [1], suggesting a positive role for the PKC family in general in platelet activation. However, calcium responses are clearly negatively regulated by PKC isoforms [2], and we have shown by pharmacological and genetic approaches that PKCδ is a negative regulator of platelet aggregation by modulating actin dynamics through VASP [3], [4]. Individual PKC isoforms therefore play distinct roles, both positive and negative, during platelet activation, and the effect of broad-spectrum PKC inhibition or activation reflects a balance of effects on positive and negative regulatory pathways [1].

Human platelets express predominantly four PKC isoforms: α, β, δ and θ. In addition to these, mouse platelets express PKCε [3]–[9]. The specific importance of each isoform is hard to assess by pharmacological approaches owing to the lack of isoform specificity of these agents. The availability of biochemical and genetic tools has allowed the functions of specific isoforms to be addressed. Using such approaches, we and others have recently demonstrated highly specific roles for individual PKC isoforms in regulating platelet function: PKCα is critically required for granule secretion and secretion-dependent aggregation [10], [11]; PKCβ is recruited to integrin α_IIb_β_3_ and positively regulates outside-in signalling [12]; PKCδ, in contrast, negatively regulates filopodia formation, and lack of PKCδ leads to enhanced platelet aggregation [13].

PKCθ is a novel (i.e. DAG-sensitive, Ca^2+^-insensitive) PKC isoform, predominantly expressed in T-cells, muscle cells and platelets [14], [15]. PKCθ^−/−^ mice exhibit reduced T cell activation, proliferation and IL-2 production downstream of T-cell receptor stimulation, owing to markedly reduced activation of multiple transcription factors [16], [17] and, as a result, these mice are resistant to some models of autoimmune disease [18]–[20]. PKCθ may also regulate fat-induced insulin resistance [21]. Selective PKCθ inhibitors are therefore of great clinical interest [22], [23], although none of those currently in development have yet become commercially available.

We have previously shown that PKCθ is physically associated with, and phosphorylated by, the tyrosine kinase, Btk [4]. However, lack of available PKCθ-selective inhibitors has curtailed research on the role of this isoform in human platelets. Shattil and co-workers have reported PKCθ-deficient platelets spread poorly on fibrinogen, suggesting that PKCθ positively regulates outside-in signalling. In addition, they demonstrated that PKCθ does not regulate platelet activation in response to a Gq/Gi coupled agonists PAR4 agonist or to ADP [24]. However, this study did not examine the role of PKCθ in collagen-induced platelet activation.

Given the primary role played by collagen in inducing platelet activation during the very early stages of thrombosis, and the parallels between signalling downstream of the collagen receptor GPVI and that downstream of immunoreceptors, it was now important to determine the role played by PKCθ in collagen-induced platelet activation and thrombus formation. We report that PKCθ negatively regulates GPVI-dependent α-granule secretion and integrin α_IIb_β_3_ activation and thereby is the only PKC isoform yet described with this function. Furthermore, loss of this negative regulation in PKCθ^−/−^ platelets leads to enhanced thrombus formation under flow *in vitro*. These results reveal a novel negative regulatory pathway in platelet activation, and have relevance to the current clinical and pharmaceutical interest in PKCθ inhibitors.

## Methods

### Materials

Unless stated, all reagents were from Sigma Aldrich (Poole, Dorset, U.K.). Cross-linked collagen-related peptide (CRP) was from Professor Richard Farndale (Biochemistry, University of Cambridge, U.K.). Horm collagen was from Axis Shield (Bicton, Cambs., U.K.). Phycoerythrin (PE)-labelled JON/A and fluorescein isothiocyanate (FITC)-labelled Wug.E9 (anti-P-selectin) antibodies were from Emfret Analytics (Eibelstadt, Germany). Anti-PKCα, -PKCβ, -PKCδ, -PKCε and anti-tubulin antibodies were from BD Transduction Laboratories (Oxford, U.K). Anti-PKCθ antibody was from Cell Signaling Technology (New England BioLabs, Hitchin, U.K.). horseradish-peroxidase (HRP)-conjugated anti-mouse IgG and anti-rabbit IgG secondary antibodies, and enhanced chemiluminescent (ECL) reagents were from Amersham (Little Chalfont, Bucks., U.K.). Luciferin-luciferase reagent was from Chronolog (LabMedics, Manchester, U.K.).

### Washed platelet preparation

PKCθ^−/−^ C57BL6/J mice have been described previously [17]. Wildtype C57BL6/J mice were used as control. Use of mouse platelets was approved by local research ethics committee at the University of Bristol, U.K. and mice were bred for this purpose under UK Home Office licence (PPL 30/2386) held by AWP. Washed platelets were prepared as previously described [13]. Of note, platelets were treated with indomethacin (10 µM). Platelets were rested for 30 min after centrifugation.

### Electrophoresis and Western blotting

Washed platelets (2×10^8^/ml) were lysed in Laemmli sample buffer. Proteins were resolved by electrophoresis in 9% SDS-polyacrylamide gels. Samples were then transferred to polyvinylidene difluoride membranes, blocked with 10% bovine serum albumin, and subjected to immunoblotting with specific antibodies to various PKC isoforms, as described in the text. Primary antibody binding was detected by HRP-conjugated secondary antibodies are revealed using ECL reagents.

### Aggregation

Washed platelets (2×10^8^/ml) were stimulated by CRP or collagen in an aggregometer (Chrono-Log, Labmedics, Manchester, U.K.) at 37°C, under continuous stirring at 1000 rpm. Aggregation was monitored by optical turbidometry.

### Dense granule secretion

ATP release from dense granules was monitored using Chrono-Lume luciferin-luciferase reagent according to the manufacturer's instructions.

### Analysis of α_IIb_β_3_ activation and α-granule secretion by flow cytometry

Washed platelets (4×10^7^/ml) were aliquoted into tubes containing optimal concentrations of PE-JON/A or FITC-anti-CD62P, which bind to active integrin α_IIb_β_3_ and surface-exposed P-selectin (CD62P), respectively, and CRP at the final concentrations indicated, for 15 min. Analysis of 20,000 events was performed using a Becton Dickinson FACScan. The platelet population as identified by forward and side scatter profile. Data were analysed using WinMDI version 2.8.

### DIC imaging of platelet adhesion and spreading

Measurement of static platelet adhesion and spreading was performed as previously described [13]. Glass coverslips were coated with fibrinogen, CRP or collagen and mounted in a live-cell chamber. Adhesion and spreading of washed platelets (2×10^7^/ml) was followed by differential interference contrast (DIC) microscopy with a wide-field microscope DM IRB attached to an ORCA ER camera (63x/1.40 NA oil objective) (Leica Microsystems, Milton Keynes, UK). Images were processed with OpenLab 4.03 (Improvision). The surface area of adherent platelets was measured using Volocity software (Improvision), while the number of adherent platelets was counted manually.

### In vitro thrombus formation

Flow-induced thrombus formation was assessed basically as described before [25]. A Leica wide-field microscope DM IRB (63x/1.40 NA oil objective), attached to an ORCA ER camera was used for image capture (Leica Microsystems, Milton Keynes, UK). Heparin/PPACK-anticoagulated mouse blood was flowed over immobilised collagen through a parallel plate perfusion chamber, at a fixed shear rate of 1000 s^−1^ for 4 minutes. For each experiment, at least 10 random phase-contrast images were captured, which were then averaged. Recorded images were analyzed with ImagePro software.

### Statistics

Statistical analyses were performed using GraphPad Prism software, unless stated otherwise, using two-way ANOVA with Bonferroni post-test; p<0.05 was considered significant. Bar charts show mean data±SEM (where ‘n’ denotes the number of individual mice used).

## Results

### PKCθ^−/−^ platelets exhibit normal expression of other PKC isoforms

**Figure 1:**
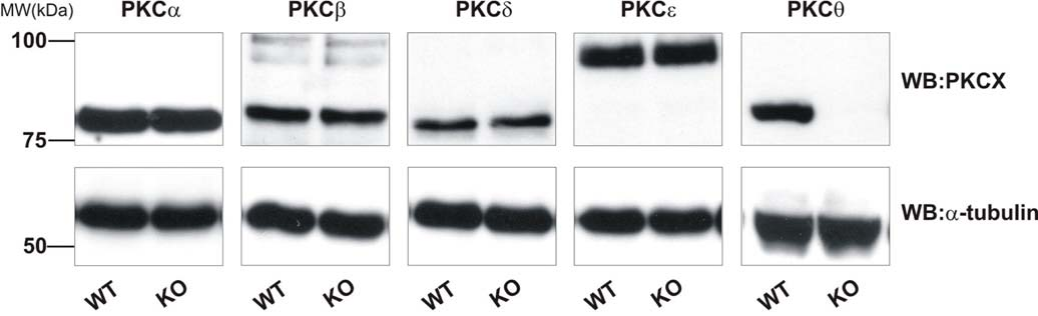
PKC isoforms are not upregulated in PKCθ^−/−^ mice. Platelets lysates from wild-type (WT) or PKCθ^−/−^ (KO) mice were assessed for PKC isoform expression by SDS-PAGE and western blotting using specific antibodies for PKCα, -β, -δ, -θ and -ε. Membranes were stripped and re-probed for α-tubulin as indicated to ensure equal loading of protein. Blots are representative of three independent experiments.

In order to be confident that any differences seen between PKCθ^−/−^ and wild-type (WT) platelets were due to loss of PKCθ, and not due to altered expression of other PKC isoforms, we assessed the expression of the major PKC isoforms in platelets by western blotting. In addition to PKCθ, mouse platelets strongly express PKCα, -β, -δ, and -ε. No difference in expression of these isoforms was seen in PKCθ^−/−^ platelets relative to WT platelets (Fig. 1). The blotting membranes were stripped and re-probed for α-tubulin, to ensure equal loading of proteins between samples (Fig. 1, lower panels).

### PKCθ has a small positive effect on platelet spreading on fibrinogen

**Figure 2:**
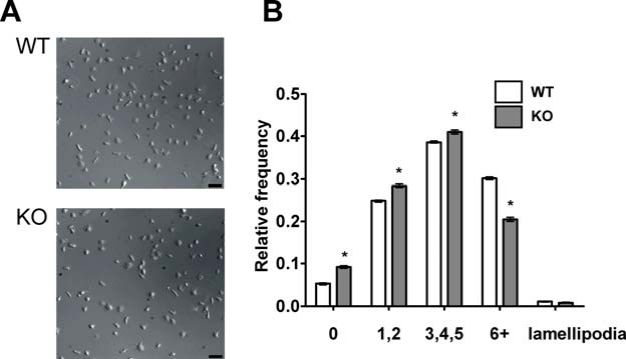
PKCθ positively regulates filopodia number when platelets spread on fibrinogen. Platelets were deposited on fibrinogen-coated coverslips in a live-cell chamber for 45 min and visualized by DIC microscopy. Five fields of view were selected at random, and one such field is shown in (*A*) for WT and PKCθ^−/−^ platelets. In (*B*), filopodia number was counted for each visible platelet and the number of platelets with none, few (1–2), some (3–5) or many (6 or more) filopodia were expressed as a proportion of the total number of platelets in view. Shown are combined data from three independent experiments. Bar indicates 10 µm.

Others have reported that platelet spreading on fibrinogen was partially defective in PKCθ^−/−^ platelets [24]. We were able to confirm and extend this result, demonstrating that both adhesion of platelets and specifically the degree of filopodia generation, rather than lamellipodia, 45 minutes after static deposition on fibrinogen-coated coverslips, were reduced in PKCθ^−/−^ platelets (Table 1). We analysed the kinetics of the spreading process to determine any further qualitative differences in spreading. Platelets during/after spreading were scored for number of filopodia and categorized as having none, few (1 or 2), some (3, 4 or 5) or many (6 or more) filopodia. The relative frequency of each morphology was determined and is shown in Fig. 2. 45 minutes after deposition on the coverslip, most WT platelets had formed at least a few filopodia, although very few platelets formed lamellipodia, consistent with other reports [26]. PKCθ^−/−^ mice had a significantly different distribution of filopodial number, with a lower proportion forming 6 or more filopodia (p<0.001). As a consequence, a greater proportion of PKCθ^−/−^ platelets formed 0, 1 or 2 filopodia than WT platelets (p<0.001). Thus, PKCθ has a small, positive regulatory role in filopodia generation on fibrinogen.

**Table 1:** PKCθ does not regulate adhesion or spreading on CRP or collagen.

	Adhesion			Surface area (µm^2^)		
	WT	KO		WT	KO	
Fibrinogen	111.1±4.5	82.7±12.0	*	24.9±1.9	21.6±0.9	*
CRP	83.7±7.3	79.6±2.2	ns	25.8±1.0	26.5±1.7	ns
Collagen	130.4±34.6	137.2±41.8	ns	14.5±0.6	14.4±0.8	ns

Platelets were deposited on fibrinogen, CRP or collagen-coated coverslips in a live-cell chamber for 45 min and visualized by DIC microscopy. Five fields of view were selected at random and the number of adherent platelets was counted (adhesion) and spread surface area measured. Adhesion is total number of platelets adherent to the surface within a single 1000 µm^2^ field of view. Shown are combined data from three independent experiments (mean±SEM; ^*^ indicates p<0.05; ns = not significant).

### PKCθ does not regulate adhesion or spreading on CRP or collagen

Since PKCθ had a role in platelet adhesion and spreading on fibrinogen, its role in adhesion and spreading on CRP and collagen was also assessed. CRP is a selective GPVI agonist, whereas collagen activates both GPVI and integrin α_2_β_1_. In contrast to fibrinogen, no significant effect was seen on adhesion or total platelet surface area on either of these substrates (Table 1). Platelet interaction with collagen is therefore not affected by absence of PKCθ.

### PKCθ negatively regulates CRP-induced platelet activation

We further investigated whether PKCθ regulates platelet activation following GPVI stimulation. Activation of GPVI leads to secretion of α-granules and dense granules, and activation of integrin α_IIb_β_3_. The latter is known as inside-out signalling and is necessary for platelet aggregation.

**Figure 3:**
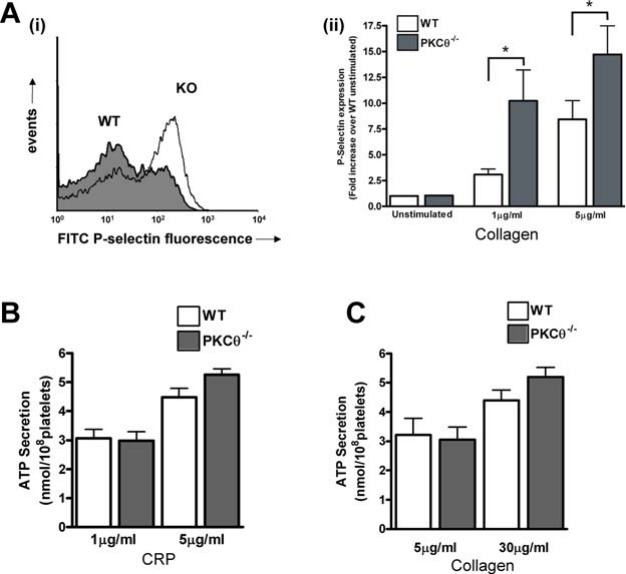
PKCθ negatively regulates α-granule secretion. *A:* Washed platelets from WT or KO mice were stimulated with CRP (1 or 5 µg/ml) in the presence of FITC-labelled anti-P-selectin antibody for 15 minutes, and surface labelling measured by flow cytometry. Representative histograms are shown in *A(i)* (1 µg/ml), and fold-increase in geometric mean compared to unstimulated platelets is shown in *A(ii)* (mean±SEM; n = 8). *B, C*: ATP secretion from dense granules in response to CRP (*B*) or collagen (*C*) was monitored in a luminometer using the luciferin-luciferase reaction. Data are presented as mean±SEM (n = 4).

CRP-induced surface expression of P-selectin, a marker of α-granule release was enhanced in the absence of PKCθ. In WT platelets, 1 µg/ml CRP induced a 3.1±0.5 –fold increase over basal in FITC-P-selectin fluorescence, which was increased to 10.2±3.0 –fold in PKCθ^−/−^ platelets (n = 8; p<0.05; Fig. 3A), suggesting that PKCθ negatively regulates the release of these granules. Interestingly, however, no difference in ATP secretion was seen between PKCθ^−/−^ and WT platelets in response to CRP (Fig. 3B) or collagen (Fig. 3C).

**Figure 4:**
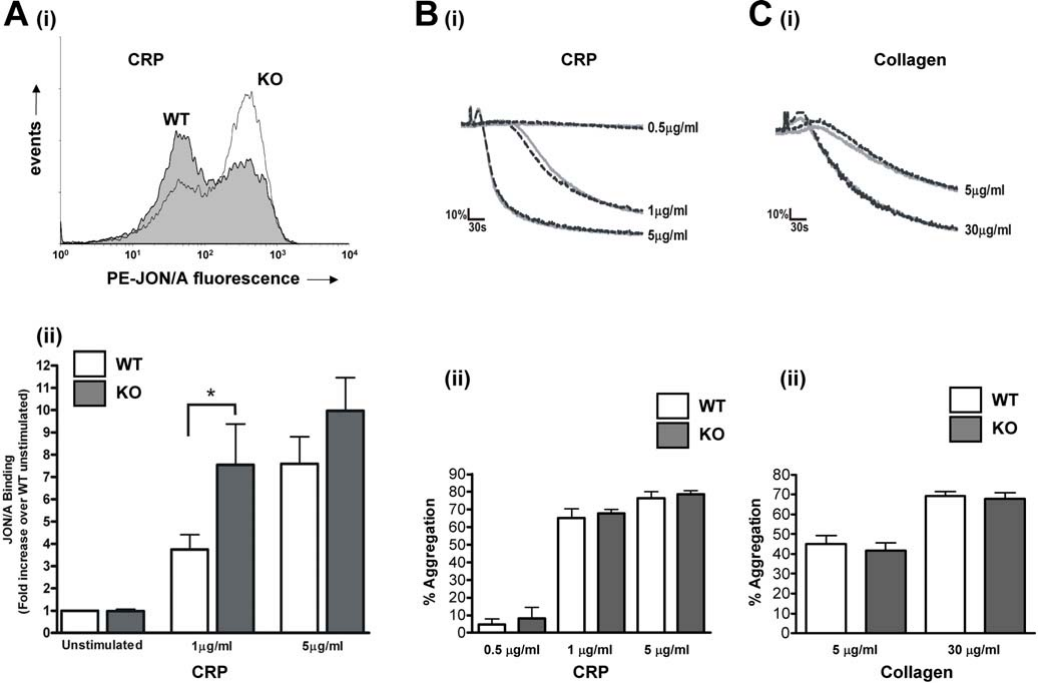
PKCθ negatively regulates CRP-stimulated integrin α_IIb_β_3_ activation but not platelet aggregation. *A*: Washed platelets from WT or KO mice were stimulated with CRP (1 or 5 µg/ml) in the presence of PE-labelled JON/A for 15 minutes, and surface labelling measured by flow cytometry. Representative histograms are shown in *(i)* (1 µg/ml), and fold-increase in geometric mean compared to unstimulated platelets is shown in *(ii)* (mean±SEM; n = 8). *B, C*: Platelet aggregation in response to CRP (*B*) or collagen (*C*) was monitored by turbidometry. Traces, shown in *B(i)* and *C(i)*, are representative of at least three separate experiments. Mean extent of aggregation at 5 min (±SEM; n = 3) is shown in *B(ii)* and *C(ii)*, for CRP and collagen, respectively.

α_IIb_β_3_ activation was determined by flow cytometry using JON/A, an antibody that recognises the active conformation of this integrin. Importantly, JON/A binding was almost doubled in platelets activated by 1 µg/ml CRP, from 3.8±0.7 –fold over basal in WT to 7.5±1.8-fold in PKCθ^−/−^ platelets (n = 8; p<0.05; Fig. 4A). In contrast, a higher concentration of CRP (5 µg/ml) was not significantly affected (7.6±1.2 –fold in WT compared to 10.0±1.5 –fold in PKCθ^−/−^; n = 8; p = 0.81; Fig. 4A). These data suggest that PKCθ negatively regulates GPVI-dependent α_IIb_β_3_ activation, but that at high concentrations this inhibition can be overcome. Interestingly however, platelet aggregation was not affected at either of these concentrations of CRP (Fig. 4B), nor was collagen-induced aggregation affected (Fig. 4C).

### PKCθ negatively regulates thrombus formation *in vitro*

**Figure 5:**
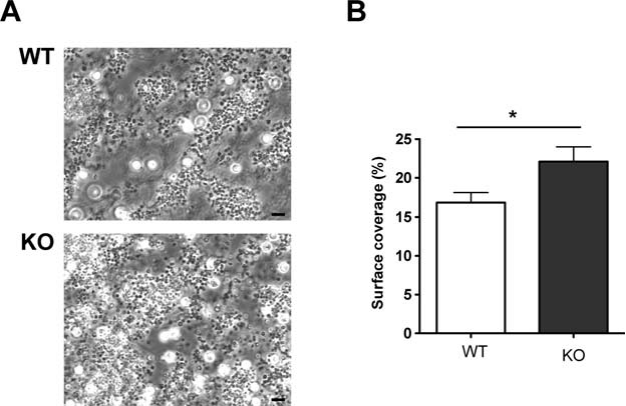
PKCθ negatively regulates thrombus formation on collagen under flow *in vitro*. Whole blood was passed over a collagen-coated coverslip at 1000 s^−1^ for four minutes then observed by phase contrast microscopy (*A*). Surface coverage was measured and is shown in *B* as mean±SEM for three independent experiments. Bar indicates 10 µm.

Since PKCθ^−/−^ aggregated normally, despite increased α_IIb_β_3_ activation and α-granule secretion, we investigated whether the role of PKCθ might become more apparent during thrombus formation in the more physiological setting of flow conditions. Anticoagulated whole blood was passed over a collagen-coated coverslip through a parallel-plate flow chamber at a shear rate of 1000 s^−1^, and thrombi observed under phase contrast after 4 min. Figure 5 shows that platelets from WT mice formed substantial thrombi on the collagen surface, However, platelets from PKCθ^−/−^ formed significantly larger thrombi, suggesting that the negative role of PKCθ is necessary to restrict thrombus size under flow conditions.

## Discussion

PKC activation is generally considered to positively regulate platelet signalling, since platelet activation is inhibited by broad-spectrum PKC inhibitors, and PKC activators can enhance platelet activation. However, here we show that the role of PKCθ is more complicated than this, as it negatively regulates α-granule secretion and inside-out signalling to integrin α_IIb_β_3_, yet positively regulates outside-in integrin signalling. In the absence of PKCθ, thrombus formation was markedly enhanced, suggesting that PKCθ restricts thrombus size.

First, we observed a significant reduction in PKCθ^−/−^ platelet adhesion and reduced spreading on fibrinogen compared to WT platelets, in agreement with Soriani et al. [24]. Interestingly, Soriani's study showed an approximately 50% reduction in spread platelet surface area whereas our study only showed a 13% reduction. This apparent quantitative (though not qualitative) discrepancy could result from technical differences between our experiments. We used DIC microscopy to image platelet spreading, and the surface area of platelets was measured by manually outlining each cell (approximately 25 µm^2^). Another study by McCarty et al [26] that used this approach saw a similar surface area. In both McCarty's study and ours, mouse platelets rarely formed large lamellipodia when spreading on fibrinogen, (in contrast to human platelets, which form full lamellipodia on fibrinogen) and filopodia were still apparent even after 45 minutes. In contrast, Soriani et al. [24] measured the surface area by confocal microscopy of rhodamine-phalloidin stained platelets, and reported a much lower surface area (approximately 8 µm^2^). Rather than measure the surface area directly, this method measures F-actin coverage, perhaps suggesting that PKCθ regulates actin polymerization. WT platelets spread on fibrinogen and imaged using this method do not appear to exhibit the spiky morphology we and others [26], [27] observe using DIC microscopy. Our analysis suggests that PKCθ positively regulates filopodia formation, since a smaller proportion of PKCθ^−/−^ platelets showed many (>5) filopodia compared to WT. Regardless of these quantitative differences, both of our studies qualitatively agree that PKCθ is a positive regulator of outside-in signalling by integrin α_IIb_β_3_.

In contrast, PKCθ negatively regulates GPVI-induced α_IIb_β_3_ activation. The selective GPVI agonist, CRP, induced a concentration-dependent increase in binding of JON/A, an activation state-specific α_IIb_β_3_ antibody. In PKCθ^−/−^ platelets this was markedly enhanced compared to WT at an intermediate concentration of CRP, though not at a higher concentration, suggesting that PKCθ reduces expression of active α_IIb_β_3_ on the platelet surface, although inhibition can be overcome as agonist stimulation increases. It has been previously reported that PKCθ does not regulate α_IIb_β_3_ activation in response to ADP or AYPGKF [24], both of which act through G protein-coupled receptors, suggesting that the regulatory role of PKCθ may be specific to GPVI signalling.

CRP-induced aggregation was not affected by the absence of PKCθ, however. Similarly, collagen-induced aggregation was also the same in WT and PKCθ^−/−^ platelets. The lack of any effect on the rate or extent of aggregation was surprising, especially in response to 1 µg/ml CRP. At this concentration, the rate of aggregation was submaximal and yet the extent of integrin activation strongly enhanced. It might be expected, therefore, that the increased integrin activation would accelerate aggregation. However, since the extent of aggregation in response to 1 µg/ml CRP was almost maximal, further enhancement of α_IIb_β_3_ in PKCθ^−/−^ platelets can have little further effect. The apparent disparity between absolute levels of integrin activation and extent of aggregation highlights the large level in integrin reserve believed to exist in platelets. β_3_^+/−^ platelets, with only 50% of the WT levels of β_3_ on their surface, have almost identical bleeding times and aggregation responses to PMA, ADP, thrombin and arachidonic acid compared to WT platelets [28]. In like manner, although WT platelets show approximately 50 % less integrin activation than PKCθ^−/−^ platelets at 1 µg/ml CRP, we should not expect this necessarily to relate to a difference in the extent of aggregation.

PKCθ also negatively regulates α-granule secretion, although no difference in dense granule secretion was observed. This suggests that the release of different platelet granules is regulated by distinct mechanisms. The PKC family in general is a critical positive regulator of platelet granule secretion [2], [10], [11], although this positive function is likely to be mediated through conventional (Ca^2+^-dependent) isoforms [5], [10], [11]. Thus, it appears that the different PKC isoforms have contrasting roles in platelet α-granule secretion: PKCα is critically required for α-granule secretion, and PKCθ acts to counter this action. PKCα is also critically important for dense granule secretion, which is not countered by PKCθ. It has been suggested that PKCδ, closely related to PKCθ, may negatively regulate GPVI-dependent dense granule secretion [5]. This interpretation was based on the use of rottlerin, a supposedly specific PKCδ inhibitor (though several PKCδ-independent targets have been reported [29]–[31]). However, we have previously reported that rottlerin enhances GPVI-dependent dense granule release even in PKCδ^−/−^ mice [13]. Thus, negative regulation of GPVI-dependent dense granule release does not appear to be mediated by either PKCδ or PKCθ.

PKCθ negatively regulates thrombus formation under flow over a collagen-coated surface. Binding to collagen activates GPVI, leading to integrin α_IIb_β_3_ activation, which is enhanced in PKCθ^−/−^ platelets. The increased number of adhesive contacts between platelets may accelerate the growth of the thrombus. Thus, negative regulation of inside-out signalling by PKCθ may be an important brake on thrombus growth at a site of injury. This effect is in contrast to the lack of effect seen in aggregation, highlighting the importance of physiological flow conditions [32]. In standard aggregometry, platelets exhibit a very large integrin reserve, whereas under flow, with higher shear force on any platelet-platelet interactions, integrin activation may be a limiting factor. Increased α_IIb_β_3_ activation would therefore enhance thrombus growth. This effect may be partially countered by the slightly reduced platelet adhesion to fibrinogen and reduced subsequent spreading, perhaps leading to fewer platelet-platelet contacts. Given the large effect on integrin activation compared to the smaller effect on spreading, however, the balance of these appears to favour increased thrombus size in PKCθ^−/−^ platelets.

In summary, we have shown that PKCθ negatively regulates GPVI-dependent inside-out signalling, in contrast to the positive role generally ascribed to the PKC family in general. Although enhanced integrin α_IIb_β_3_ activation does not lead to increased aggregation in an aggregometer tube, PKCθ^−/−^ platelets display enhanced thrombus formation on collagen under flow, suggesting that, under more physiological conditions, the regulatory role of PKCθ may restrict thrombus size. This may impact on the clinical safety of PKCθ inhibitors.
